# Antiviral and cytotoxic activities and chemical profiles of two species of *Abies nordmanniana* from Türkiye

**DOI:** 10.55730/1300-0527.3670

**Published:** 2024-03-11

**Authors:** Nazlı SARIKAHYA, Gaye SÜMER OKKALI, Furkan O. ÇÖVEN, Sevda ZEINALİ, Gülce GUCUR, A. Dila ÇAĞLAR, Ece UYAR, Ahmet C. GÖREN, Ayşe NALBANTSOY

**Affiliations:** 1 Department of Chemistry, Faculty of Science, Ege University, İzmir, Turkiye; 2 Department of Bioengineering, Faculty of Engineering, Ege University, İzmir, Turkiye; 3 Department of Biotechnology, Faculty of Science, Ege University, İzmir, Turkiye; 4 Department of Chemistry, Faculty of Basic Sciences, Gebze Technical University, Kocaeli, Turkiye

**Keywords:** *Abies*, resin, cone, cytotoxicity, antiviral activity, avian coronavirus

## Abstract

*Abies* is an important genus of the family Pinaceae, with about 50 species found in the highlands of Asia, Europe, North Africa, and North and Central America. The principal aim of the present work was to investigate the chemical content and biological potential of the resin and cone from *Abies nordmanniana* subsp. *bornmulleriana* and *Abies nordmanniana* subsp. *equi-trojani*, respectively. The flavonoid and phenolic contents of the resin and cones were evaluated using liquid chromatography-high resolution mass spectrometry (LC-HRMS). Additionally, the essential oil and fatty acid compositions were analyzed using gas chromatography-mass spectrometry (GC-MS) and gas chromatography-flame ionization detector (GC-FID), respectively. Cytotoxicity of the extracts and essential oils were screened against certain cancer cell lines, namely, human prostate adenocarcinoma cell line (PC3), human lung adenocarcinoma cell line (A549), human pancreatic cancer cell line (PANC-1), human hepatocellular carcinoma cell line (HepG2), human breast cancer cell line (MDA-MB231), and normal human lung fibroblast cell line (CCD-34-LU), with MTT [3-(4,5-dimethylthiazol-2-yl)-2,5-diphenyl-2H-tetrazolium bromide] assay. According to the MTT results, hexane extracts of both cone (CH) and resin (RH), ethanol-water (CEW), dichloromethane (CD), and acetone (CA) extracts of the cone mostly inflict cytotoxicity in HepG2 cell line. Antiviral activities of *Abies nordmanniana* subsp. extracts at doses of 5 μg/g and 10 μg/g were also evaluated in ovo for their virucidal activity against avian coronavirus. *Abies nordmanniana* subsp. extracts exhibited concentration-dependent antiviral activity on specific pathogen-free embryonated chicken eggs. Significantly, cone acetone extract (CA), cone ethanol extract (CE), and cone dichloromethane extract (CD) of *Abies nordmanniana* subsp. exhibited strong inhibition of the virus at a concentration of 10 μg/g. The most potent virucidal activity was observed with ethanol-water extract of conifer form (CEW). According to these results, it was proved that *Abies nordmanniana* species could be a potential, sustainable, and renewable drug source, especially considering the impressive antiviral and significant cytotoxic activity potentials.

## 1. Introduction

*Abies*, an important genus of the family Pinaceae [1,2], are evergreen, tall coniferous trees, containing 51 species native to the northern hemisphere. They are distributed naturally in the temperate and boreal regions of the world and are mainly found in North and Central America, Europe, North Africa, and Asia (Himalayas, South China, and Taiwan) [3,4]. This genus establishes pure or mixed forests in high mountains, lowlands, and even sea-level regions [5]. The history of the genus *Abies* dates back to the Hittites. At present, it is the most common form of traditional treatment, especially against bronchitis and shortness of breath [6]. In some regions, the resin obtained from the trunk of the tree is used as an antiseptic and wound healer, and its cones are used for many purposes in traditional medicine [7,8]. The genus *Abies* has also beneficial applications in pharmacological studies owing to its content of bioactive constituents [9]. It has been reported that 277 compounds were found in 19 *Abies* genera in the isolation studies carried out for chemical content determination since 1938. They are mostly terpenoids, triterpenoids, flavonoids, lignans, and a few other constituents including phenols, steroids, fatty acids, and fatty alcohols [9,10]. Several biological activities have also been investigated, including antimicrobial [11–13], antitumor [14–16], antibacterial [17–21], antifungal [22], antiulcerogenic [23,24], antiinflammatory [25,26], antihypertensive [23], antitussive [25,27], and effects on the central nervous system (CNS) [28,29]. Considering the diversity of use among the folk medicine of the genus *Abies*, the fundamental aim of this study is to elucidate the content of pharmacologically active compounds in the resin of *Abies nordmanniana* subsp. *bornmulleriana* from Bolu and cones of *Abies nordmanniana* subsp. *equi-trojani* from Çanakkale of Türkiye. Flavonoid, phenolic, essential oil, and fatty acid components of resin and cones were evaluated using LC-HRMS, GC-MS, and GC-FID, respectively. Cytotoxicity of the extracts and essential oils were examined against diverse cancer cell lines, namely PC-3, A-549, PANC-1, HepG2, MDA-MB-231, and healthy cell line CCD-34Lu, with MTT assay. Antiviral activities were evaluated in ovo for virucidal activity against avian coronavirus.

## 2. Materials and methods

### 2.1. Plant material

Resin samples from *Abies nordmanniana* subsp. *bornmulleriana* were collected from their natural habitats along Seben of Bolu, Türkiye (40°24′41″N, 31°34′12″E) and cone forms of *Abies nordmanniana* subsp*. equi-trojani* were collected from their growth areas Bozcaada-Bayramiç of Çanakkale, Türkiye (39°48′34″N, 26°36′43″E) in June 2021.

### 2.2. Chemicals

As a stock solution, 100 mg/L dihydrocapsaicin (97%, Sigma-Aldrich) solution was prepared freshly. The solution was used as an internal standard (IS). In the LC-HRMS analysis, the following compounds were used as standards for method validation: (−)-epigallocatechin gallate (>97% purity, TRC Canada), (−)-epigallocatechin (>97% purity, TRC Canada), chlorogenic acid (≥95% purity, Sigma-Aldrich), fumaric acid (≥99% purity, Sigma-Aldrich), (−)-epicatechin (≥90% purity, % Sigma-Aldrich), ascorbic acid (≥99% purity, Sigma-Aldrich), (−)-epicatechin gallate (>97% purity, TRC Canada), verbascoside (86.31% purity, Hwi Analytik Gmbh), caffeic acid (≥98% purity, Sigma-Aldrich), (+)-trans taxifolin (>97% purity, TRC Canada), luteolin-7-glucoside (>97% purity, TRC Canada), luteolin-7-rutinoside (>97% purity, Carbosynth limited), rosmarinic acid (≥96% purity, Sigma-Aldrich), vanilic acid (≥97% purity, Sigma-Aldrich), apigenin-7-glucoside (>97% purity, EDQM CS), dihydrokaempferol (>97% purity, Phytolab), hyperoside (>97% purity, TRC Canada), ellagic acid (>97% purity, TRC Canada), quercetin (≥95% purity, Sigma-Aldrich), quercitrin (>97% purity, TRC Canada), myricetin (>95% purity, Carl Roth GmbH + Co), scutellarein (>97% purity, TRC Canada), caffeic acid phenethyl ester (CAPE, ≥97% purity, Sigma-Aldrich), salicylic acid (≥98% purity, Sigma-Aldrich), naringenin (≥95% purity, Sigma-Aldrich), luteolin (95% purity, Sigma-Aldrich), nepetin (≥98% purity, Supelco), (−)-sinensetin (>97% purity, TRC Canada), apigenin (>97% purity, TRC Canada), hispidulin (>97% purity, TRC Canada), acacetin (>97% purity, TRC Canada), pyrogallol (≥98% purity, Biosynth), chrysin (≥96% purity, Sigma-Aldrich).

### 2.3. Preparation of extracts and essential oils

Resin samples were collected and subjected to three different extraction methods. Initially, the first set of resin samples (0.6324 g) underwent extraction with ethanol (35 mL) using a homogenizer (Silverson, L5M-A, USA) overnight at room temperature. The second set of samples (3.004 g) was subjected to water distillation for 8 h using a Clevenger-type apparatus to extract essential oils. Lastly, the third set of samples (2.996 g) was refluxed with *n*-hexane (500 mL) for 6 h using a Soxhlet apparatus. All resin extracts were concentrated under vacuum till dryness at 40 °C. The collected cone samples were dried under dark conditions, ground, and prepared for extraction. Subsequently, they were extracted three times with dichloromethane, acetone, and ethanol (10 mL each) with a homogenizer for 5 h at room temperature, separately. The extract rich in fatty acids was prepared with Soxhlet apparatus using *n*-hexane (500 mL) for 6 h. Additionally, the essential oil of samples was obtained by water-distillation with a Clevenger apparatus using 10 g of crushed dried cones. The essential oils were dried over anhydrous granular sodium sulfate. All extracts and essential oils were stored at +4 °C away from light until chemical analysis and biological activity studies.

### 2.4. Preparation of samples for LC-HRMS analysis and optimization of the method

Initially, 50–100 mg of the dried extracts was dissolved in methanol:water (60:40) in a 5 mL volumetric flask. Sonication was used to obtain a clear solution. Next, internal standard [(100 μL of dihydrocapsaicin solution), (100 mg/L of stock solution)] was added and the volume was diluted with the mobile phase. The flask was mixed gently and heated to obtain a clear solution. The clear solution was filtered via a 0.45 μm Millipore Millex-HV filter. One milliliter of the final solution was transferred into a capped autosampler vial. Injection of LC was set to take 2 μL of the sample for each run. The temperature of the autosampler was kept at 15 °C during the experiments [30–33]. LC-HRMS experiments were conducted on a Thermo ORBITRAP Q-EXACTIVE mass spectrometry, equipped with a Troyasil C18 column (150 × 3 mm i.d., 5 μm particle size). For the separation of flavonoids and phenolics, the mobile phases A and B were formed of 1% formic acid-water and 1% formic acid-methanol, respectively. The gradient of the mobile phase was programmed as 50% A and 50% B for 1.00 min, 100% B for 5 min, and finally 50% A and 50% B for 9 min. The column temperature was fixed at 22 °C while the flow rate was 0.35 mL/min. [31,34]. Environmental conditions were maintained at a relative humidity of 50 ± 15% and a temperature of 22.0 ± 5.0 °C. Acidified methanol and water gradient were found as the best mobile phase. This phase was also determined as a proper mobile phase for ionization abundance and separation of compounds. Using the ESI source, optimal ionization of small and relatively polar compounds was observed. The instrument scanned ions in the range of *m/z* 100–900 were scanned in high-resolution. Compounds were identified by comparing the retention time with standard compounds (ranging in purity from 95%–99%; see chemicals section). HRMS data were obtained from Bezmialem Vakif University, Drug Application and Research Center Library (ILMER). Dihydrocapsaicin (purity 95%) was used as an internal standard for LC-HRMS to reduce repeatability problems caused by external effects. The detailed mass parameters of each compound are given in Table S1 [30,31,34,35].

### 2.5. GC-FID analysis

Methyl esters of fatty acids were analyzed with Agilent GC-FID combined system using SUPELCO SP TM-2560 column (100 m × 0.25 mm × 0.20 μm). Each extract (5 mg) was mixed with 2 mL of 2 M potassium hydroxide in methanol and then vortexed. Following this, 2 mL of isooctane was added on the mixture and vortexed again. Subsequently, the samples were centrifuged at 3000 rpm for 4 min. Approximately 1 mL of the supernatant was vialed and 1 μL of the sample was injected into the GC-FID system. The oven temperature was programmed to start from 140 °C and increase by 4 °C every 5 min so that the final temperature reaches 240 °C and remain there for 5 min. Helium was used as carrier gas at a constant flow rate of 1 mL/min. Supelco Fame Mix 37 library data was used for the identification of compounds. The standard containing *n*-alkanes (Supelco 49452-U) was used for the calculation of relative retention indices.

### 2.6. GC-MS analysis

The essential oil analysis was carried out using HP 6890 Series GC system, equipped with an INNOWAX column (Hewlett Packard, No: 19091N-116). The column dimensions were 60 m × 0.32 mm i.d., with a 0.25 μm film thickness. The injection part temperature was set at 150 °C. The essential oils were diluted with *n*-hexane, and 1 μL of each sample was injected in the split mode with a split ratio of 50:1. The oven temperature was programmed to start at 60 °C for 4 min, followed by a ramp of 4 °C per min until reaching 230 °C, where it remained for 5 min. Pure helium was used as carrier gas, with a flow rate of 0.7 mL/min.

### 2.7. In vitro cytotoxicity assay

In vitro cytotoxicity assay screening of the extracts and essential oils based on metabolic cell viability was done using a modified MTT [3-(4,5-Dimethyl-2-thiazolyl)-2,5-diphenyl-2H-tetrazolium bromide)] assay [36] that is based on the cleavage of the yellow tetrazolium salt, which forms water-insoluble, purple formazan crystals that affect the mitochondrial reductase activity of viable cells. For this purpose, 1 × 10^5^ cells/well were seeded in 96-well plates in Dulbecco’s modified Eagle’s F12 medium (DMEM/F12) supplemented with 10% fetal bovine serum (FBS) and 0.1% penicillin/streptomycin at a volume of 100 μL per well, and incubated overnight. Following this, the extracts were dissolved in DMSO and diluted with PBS into the desired concentration (CH, CE, CD, CA, CEW, RH, RE, and REO (50, 5, and 0.5 μg/mL) and CEO (50, 5, 0.5, 1, 0.1, and 0.01 μg/mL) were added to the cells for 48 h at 37 °C. As a positive control, doxorubicin (20, 2, and 0.2 μg/mL) was used. After 48 h, the MTT assay was applied to determine cell viability. For MTT assays, 20 μL of MTT solution (from the stock concentration of 2.5 mg/mL) was added to all wells and incubated at 37 °C for 3 h. Subsequently, 150 μL of DMSO was added to all wells. Next, the optical density (OD) was measured at 570 nm. The IC_50_ value was calculated using the GraphPad Prism 8 program based on the calculated percent viability and absorbance values. The percentage of viability was determined using the following formula:


$$\%Viable\;cells=\frac{\left[(absorbance\;of\;treated\;cells)-(absorbance\;of\;blank)\right]}{\left[(absorbance\;of\;control)-(absorbance\;of\;blank)\right]}\times 100$$

### 2.8. In ovo antiviral activity assay

#### 2.8.1. Preparation of virus

Specific pathogen-free embryonated chicken eggs (SPF-ECE) between nine and eleven days old, along with 1% chicken red blood cells (RBC), were procured from the Bornova Veterinary Control Institute in İzmir, Türkiye, for the antiviral activity assay. The infectious bronchitis virus (IBV) D274 strain, initially isolated from the Netherlands, was graciously provided by Dr Fethiye Çöven [37]. The embryo infectious dose (EID_50_) of the IBV was determined by the formula originated from the Reed and Muench (1938) method [38], which is the gold standard method for calculating EID_50_. The stock virus was diluted between 10^−1^ and 10^−12^ by using phosphate-buffered saline (PBS), and each dilution was inoculated into the chorioallantoic fluid (CAF) of four SPF-ECEs. After 48 h of incubation, the CAF was collected from each egg, and a hemagglutination test was performed. The EID_50_ titer of the stock virus was determined using the Reed and Muench formula based on the number of virus-infected eggs. For this experiment, the virus solution used was diluted from the stock virus to achieve a concentration of 100 EID_50_/0.1 mL, using PBS [39].

#### 2.8.2. Inoculation of sample-virus mixture

Samples dissolved in DMSO were diluted with PBS to obtain the final concentrations of 5 μg/g and 10 μg/g. Enfluvir was dissolved in DMSO-pure water (1:9) for use as an antiviral agent and administered at a final concentration that contained less than 5% DMSO. The stock virus was mixed with the sample at a ratio of 1:1, and 0.1 mL of each mixture was injected into the chorioallantoic fluid of SPF-ECEs. After injection, SPF-ECEs were incubated for 48 h at 37 °C, with 55% humidity [40].

### 2.9. Hemagglutination (HA) assay

Following the WOAH procedure (2018), two-fold dilutions of CAF were used in the HA experiment to evaluate the viral titer. The HA assay was conducted using V-bottom 96-well microplates, and PBS was utilized for dilution [41]. To perform the test, 25 μL of PBS was first added into all wells. Subsequently, 25 μL of CAF collected from each egg separately was placed in the first column and diluted 2-fold to the last column. To have the same volume for all wells, 25 μL of sample was discarded to the last column. After 2-fold dilution, 25 μL of 1% (v/v) chicken red blood cells (RBCs) was added into all wells, and plates were incubated for around 45 min at room temperature. HA activity was determined by the presence or absence of a tear shape. Lace-like formation represents HA positivity, while tear-shaped (or button-shaped) formation is interpreted as HA negativity. The Local Ethical Committee of Animal Experiments at Ege University authorized the HA assay methodology to determine antiviral activity (Date: 2020 No: 2020-051).

## 3. Results and discussion

In this work, resin samples from the trunk of *Abies nordmanniana* subsp. *bornmulleriana* and the cone from lateral branches of *Abies nordmanniana* subsp*. equi-trojani* from Türkiye were examined separately. The samples were chosen due to their ethnobotanical features such as the folk and traditional usage of materials in certain regions. The most common traditional use of resin from *Abies nordmanniana* subsp. *bornmulleriana* is as gum in Bolu, especially against bronchitis and shortness of breath. The most common public use of the cone from *Abies nordmanniana* subsp. *equi-trojani* is the use of water vapor or jam obtained from the cones against lung diseases such as bronchitis and chronic obstructive pulmonary disease in Çanakkale.

*Abies* species have been used as traditional medicine and food ingredients due to their specific odor and biological activities. Former studies that investigated the chemical composition of *Abies* extracts have predominantly identified monoterpenes and monoterpenoids in resin and essential oil, along with triterpenoids in needles and bark [42]. Additionally, many extracts have been identified as rich sources of flavonoids and phenolics [9]. Not only the extracts but also the essential oils of these species have been studied and are widely used, especially in the treatment of colds, as well as for indigestion, venereal diseases, and lung disorders [11]. Essential oils of the genus *Abies* exhibited considerable diversity in both the composition and percentages of compounds between species and from tree to tree [43]. The chemical content of essential oils of cone and resin were examined separately in this study (Table 1). Seventeen compounds comprising 99.99% of the total oil were identified of which limonene (33.50%) and *α*-pinene (28.79%) were recognized as major constituents of cone. Limonene is popularly used for digestive problems and abdominal pain, and as a cough suppressant [44] and is considered a safe treatment option with low toxicity, since it is rapidly absorbed and metabolized without posing a mutagenic, carcinogenic, or nephrotoxic risk in the gastrointestinal tract [45]. Additionally, Hirota et al. [46] reported its use in treating respiratory tract diseases among the public. They aimed to evaluate whether it could reduce allergic airway inflammation and improve asthma symptoms, demonstrating its potent therapeutic effect on allergic airways and asthma. Limonene also offers a range of biological activities described in the literature, such as antidepressant [47], antinociceptive [48], antidiabetic [49], antiulcerogenic activity [50], and anticancer activity both in vitro and in vivo [51]. The other major compound, *α-*pinene, in the essential oil of the conifer form, especially with its woody and pine scent, is traditionally used for respiratory problems. It has a variety of biological activities, such as gastroprotective [52], insecticidal, antiinflammatory, antiviral [53], neuroprotective [54], and antifungal [55] properties. Cone essential oil composition also includes *β-*pinene (3.55%), which is the isomer of *α-*pinene. The percentage of *α*-pinene in cone essential oil is considerably higher than that of *β-*pinene. Comparison of resin and cone essential oils revealed quite different chemical contents. While the major compounds in the essential oil components of the conifer form were limonene (33.50%) and *α*-pinene (28.79%), the main components in the resin essential oil were verbenone (18.16%) and 2,5-dimethyl furan (10.79%) (Table 1). Verbenone, a natural monoterpenoid bicyclic ketone, is an essential component of essential oils [54]. Due to its pleasant aroma, it is used in perfumes, herbal teas, aromatherapy, spices, and herbal medicines. It is also used in natural pest control against insects that damage pine trees and as an antimicrobial agent [56].

The hexane extract of both cone and resin was prepared using a Soxhlet apparatus. Five different fatty acids were detected in the gum, whereas four were identified in the cones. The composition of these fatty acids is as follows: oleic acid (41.00%), palmitic acid (20.81%), linoleic acid (15.66%), stearic acid (14.24%), and behenic acid (8.28%). In coniferous form, these amounts change as oleic acid (55.74%), palmitic acid (20.83%), stearic acid (12.49%), and linoleic acid (10.94%) (Table 2). Oleic acid is the most abundant compound for both gum (41.00%) and cone (55.74%) extracts. Oleic acid is known for both its antiviral and cytotoxic activities [57]. The antitumor activity of oleic acid has been investigated by Venepally et al. (2017), and it is shown that oleic acid is the most promising fatty acid against A-549, PC-3, MDA-MB-231, HepG2 [58]. In addition, it is known that oleic acid has an antiinflammatory effect by reducing adhesion molecules, reducing autoimmune disorders, lowering blood pressure, and reducing the risk of cancer [59]. The evident variation of content was also observed in the amount of linoleic acid. Besides, behenic acid was detected only in the hexane extract of resin. Differentiation of the components of these two extracts caused differences in their cytotoxic activities in some cell lines. In PANC-1 cell lines, the IC_50_ value could not be detected in resin hexane extract, while this value was 48.6 ± 5.28 in cones. The main difference was observed in the HepG2 cell line. Both extracts showed more activity compared to the control group doxorubicin, while the activity of the resin (17.725 ± 2.09) extract was better than that of the cones (36.67 ± 3.68). Samra et al. (2021) tested the activity of behenic acid against the HepG2 cancer line and proved that it showed potential cytotoxic activity compared to doxorubicin [60]. The fact that the resin is more active against HepG2 cell lines may be related to its behenic acid content.

In vitro cytotoxicity assays were performed based on metabolic cell viability using the MTT test which is still one of the most useful and well-liked viability assays. The MTT assay uses mitochondrial reductase to convert the water-soluble yellow dye MTT into an insoluble purple formazan. The concentration of the formazan is then evaluated by optical density at 570 nm after it has been solubilized [28]. Doxorubicin was used as a positive control to evaluate the adequate cytotoxic dose of the samples on cancerous cell lines (Figures S1–S6).

IC_50_ values of all *Abies nordmanniana* subsps. were calculated for cells in the measurable range (0.5–50 μg/mL). The percent vitality graph according to the MTT test result is presented in Table 3. According to the results, none of the samples inflicted cytotoxic effects on CCD-34Lu, MDA-MB-231, PC-3, and A-549 cell lines even at the highest concentration of 50 μg/mL. However, most of them (CH, RH, CEW, CD, and CA) had a cytotoxic effect on HepG2 cell line. The IC_50_ value of RH (17.725 ± 2.09 μg/mL) in HepG2 cell line was found to be nearly twice as cytotoxic when compared to doxorubicin (36.85 ± 0.02 μg/mL). Additionally, only CH at a concentration of 48.6 ± 5.28 μg/mL showed a cytotoxic effect on PANC-1 cells. As the healthy cell line model, we used CCD-34Lu cell line to evaluate the samples’ cytotoxic side effects on them. Based on the results, various samples did not exhibit any cytotoxic influence on CCD-34Lu cell line, suggesting that *Abies nordmanniana* subsp. may contain potential candidate molecules for future cancer studies (Figures S7–S12).

The phenolic and flavonoid contents of resin ethanol extract, cone ethanol, ethanol-water (1:1), dichloromethane, and acetone extracts were determined using LC-HRMS (Table 4). In the ethanol extract of resin, eleven different phenolics and flavonoids were determined. The major compound was fumaric acid (57050.00 mg/kg), which was not detected in any extracts of the conifer form. Following fumaric acid, other common compounds in resin ethanol extracts were salicylic acid (282.45 mg/kg) and rosmarinic acid (158.69 mg/kg), respectively.

Ethanol-water extract of the cone has the most diverse chemical contents, with eighteen phenolics and flavonoids. The most abundant of these were ascorbic acid (2508.50 mg/kg), followed by vanillic acid with 1691.89 mg/kg and quercitrin with 283.28 mg/kg. The seventeen different compounds were detected in the ethanol extract of the cone and the major compound was detected as vanillic acid (1152.93 mg/kg). When ethanol and ethanol-water extracts were compared, no significant change was observed in the amount of vanillic acid, but the presence of water had a significant effect on the increase of the amount of ascorbic acid, a type of water-soluble vitamin. The most abundant phenolics in the dichloromethane extract of the cone were rosmarinic acid (130.13 mg/kg), salicylic acid (103.91 mg/kg), and caffeic acid (38.10 mg/kg). Cone acetone extract had the most diverse content of eighteen compounds, just like the ethanol-water extract, but they were quite different from each other in quantity and content. The quantity of the major compound, vanillic acid, was 1554.36 mg/kg, and salicylic acid and ascorbic acid followed it with 352.66 mg/kg and 56.54 mg/kg, respectively.

Antiviral activity against the avian coronavirus was used to evaluate the antiviral potential of cone and resin extracts. After 48 h of incubation of SPF-ECEs with the virus-extract, embryos were examined and CAF was collected to perform the HA assay. Egg mortality, mortality (%), and HA titer (log_2_) are shown in Table 5 and Figure. The virucidal antiviral activity of several *Abies nordmanniana* subsp. samples at doses of 5 μg/g and 10 μg/g were assessed. When comparing SPF-ECEs groups, extracts of *Abies nordmanniana* subsp. displayed concentration-dependent antiviral activity. In the virus control group, there was only one dead embryo among all SPF-ECEs, as shown by the daily viability check of the embryos (Table 5) (Figure S13). The cause of death may be attributed to some injection manipulation during virus inoculation, considering the healthy status of embryos in the virus control group. The HA titer of virus control was calculated as 2048, which means the virus was replicating. Based on the results, enfluvir reduced the log_2_ HA titer compared to the control group dose-dependently by 1 log_2_ HA titer at 10 μg/g. The acetone extract of the cone (CA) exhibited virucidal activity by reducing the log_2_ HA titer at 10 μg/g, but when the embryos were investigated there was also one dead embryo in the four tested SPF-ECE’s. Remarkably, the cone dichloromethane extract (CD) and cone ethanol extract (CE) showed significant inhibition of the virus at a concentration of 10 μg/g, without having any toxicity effect on the SPE-ECEs. Both extracts reduced the HA titers 10-fold based on log_2_ HA titers at 10 μg/g. The most effective HA titer inhibition was observed in ethanol-water extract of conifer form (CEW). The sample decreased the virus activity in comparison with virus control at both concentrations of 5 μg/g and 10 μg/g. However, the concentration of 10 μg/g exhibited toxicity on embryos with three dead embryos in a group of four eggs, which means a high concentration of CEW can be toxic while a low concentration is nontoxic for chicken embryos. The rest of the extracts had slight virucidal effects on the SPE-ECEs with an average reduction of 2-fold based on log_2_ HA titers at 5 μg/g and 10 μg/g. The most abundant component in the cone ethanol-water extract (CEW) was ascorbic acid. Ascorbic acid (vitamin C) in high doses is proven to be virucidal. Vitamin C reduced the viral load of Ebstein-Barr virus (EBV)-infected cells, based on an experimental model [61]. The protective and mitigating effects of it against the virus may have been beneficial in decreasing the virus activity [62]. In some in vivo studies, the contradictory ability of vitamin C to generate reactive oxygen species through the reduction of transition metals is highlighted. The significant toxicity of CEW in SPF-ECEs at a concentration of 10 μg/g can be attributed to the potential of vitamin C to generate reactive oxygen species. Despite the data based on in vitro studies, there is a lack of clinical evidence to support the theory which suggests that vitamin C may be effective in reducing viral load. The use of vitamin C as an additional therapy for serious infections caused by the flu, RSV, herpes, and other prevalent viral disorders must be thoroughly evaluated in clinical trials [61]. Dichloromethane, acetone, and ethanol extracts have a considerable amount of salicylic acid. In 2020, Geiger et al. proved that salicylic acid interferes with viral replication of SARS-CoV-2 [63]. This suggests that salicylic acid, a metabolite of the acetylsalicylic acid (the active compound of Aspirin), may have a potential virucidal effect against IBV D274. The main chemical content difference between dichloromethane, ethanol, and acetone extracts is fumaric acid. While fumaric acid was observed as the main component with the highest percentage in the ethanol resin extract, it was not detected in the other extracts. Considering its synergistic effect with salicylic acid, fumaric acid may have suppressed the antiviral effect of salicylic acid. According to in ovo results in our laboratory, enfluvir did not exhibit the highest antiviral activity against IBV. This might be explained by the fact that the majority of antivirals only work against specific viruses. In conclusion, three *Abies nordmanniana subsp*. samples, CD, CA, and CE had significant virucidal effects on SPF-ECEs at the concentration of 10 μg/g. While CEW had the most potent antiviral effect against the virus, 10 μg/g concentration of the extract displayed a damaging effect on SPF-ECEs according to dose-dependent fatalities. The in ovo antiviral activity results suggested that *Abies nordmanniana* subsp. samples had strong virucidal effects against the avian coronavirus strain D274 of the infectious bronchitis virus (IBV). However, because most antivirals are exclusively effective against a certain virus, we specifically want to draw your attention to the findings. Further and extended studies, which include testing the virucidal effects of the extracts against different viruses, will provide a broader perspective on the virus inhibition mechanism of *Abies nordmanniana* subsp. extracts. It is important to prioritize research on natural products since the need for fast produced natural-derived supplements is proven with the emergence of SARS-Cov-2. With further research, the understanding of natural sources will increase and the production of naturally derived products will be accelerated.

## 4. Conclusions

*Abies nordmanniana* spp. has been widely used as a traditional product among the public since ancient times. For the treatment of viral diseases and cancer, which are some of the biggest problems of recent years, plants and the products obtained from them have become an undeniable option in the search for various sources and potential drugs [64,65]. In this study, it was proved that *Abies nordmanniana* spp. could be a potential drug source, especially considering the impressive antiviral activity of the green product ethanol-water extract and the significant cytotoxic activity of the fatty acid extract against HepG2 cell lines compared to the control group doxorubicin. The evergreen and perennial nature of the cones and resin of this tree also make it a great potential source for sustainable products.

## Supplementary Data

**Table S1:** Mass parameters and linear regression equation of compounds^a^–^b^.

Compounds	Molecule formula	m/z	Ionization mode	Linear interval	Linear regression equation	LOD/LOQ	R²	Recovery
Ascorbic acid	C _6_H _8_O _6_	175.0248	Negative	0.5–10	y=0.00347x-0.00137	0.39/1.29	0.9988	96.2
(−)-Epigallocatechin	C _15_H _14_O _7_	307.0812	Positive	0.3–5	y=0.00317x+0.000443	0.17/0.57	0.9947	102.22
(−)-Epigallocatechin gallate	C _22_H _18_O _11_	459.0922	Positive	0.3–7	y=0.00182x+0.000026	0.1/0.33	0.9989	94.76
Chlorogenic acid	C _16_H _18_O _9_	353.0878	Negative	0.05–10	y=0.00817x+0.000163	0.02/0.06	0.9994	96.68
Fumaric acid	C _4_H _4_O _4_	115.0037	Negative	0.1–10	y=0.00061x-0.0000329	0.05/0.17	0.9991	97.13
(−)-Epicatechin	C _15_H _14_O _6_	289.0718	Negative	0.05–10	y=0.0172x+0.0002269	0.01/0.03	0.9993	95.66
(−)-Epicatechin gallate	C _22_H _18_O _10_	441.0827	Negative	0.05–10	y=0.00788x-0.0001875	0.01/0.03	0.9995	96.54
Verbascoside	C _29_H _36_O _15_	623.1981	Negative	0.1–10	y=0.00758x+0.000563	0.03/0.1	0.9995	96.19
Caffeic acid	C _9_H _8_O _4_	179.0350	Negative	0.3–10	y=0.0304x+0.00366	0.08/0.27	0.9993	94.51
(+)-t *rans* taxifolin	C _15_H _12_O _7_	303.0510	Negative	0.3–10	y=0.0289x+0.00537	0.01/0.03	0.9978	91.66
Luteolin 7-glucoside	C _21_H _20_O _11_	447.0933	Negative	0.1–7	y=0.0162x+0.00226	0.01/0.03	0.9961	96.31
Rutin	C _27_H _30_O _16_	609.1461	Negative	0.05–10	y=0.00329x-0.00005576	0.01/0.03	0.999	96.97
Rosmarinic acid	C _18_H _16_O _8_	359.0772	Negative	0.05–10	y=0.00717x-0.0003067	0.01/0.03	0.9992	99.85
Dihydrokaempferol	C _15_H _12_O _6_	287.0561	Negative	0.3–7	y=0.0756x+0.0118	0.01/0.03	0.995	95.37
Apigenin 7-glucoside	C _21_H _20_O _10_	431.0984	Negative	0.3–7	y=0.0246x+0.00306	0.01/0.03	0.9962	96.07
Ellagic acid	C _14_H _6_O _8_	300.9990	Negative	0.05–10	y=0.0085x-0.000612	0.03/1	0.9994	101.49
Quercitrin	C _21_H _20_O _11_	447.0933	Negative	0.05–10	y=0.0179+0.0003331	0.01/0.03	0.999	97
Myricetin	C _15_H _10_O _8_	317.0303	Negative	0.1–10	y=0.0202x+0.00165	0.01/0.03	0.9993	100.1
Scutellarein	C _15_H _10_O _6_	285.0405	Negative	1+7	y=0.028x+0.00845	0.01/0.03	0.9983	95.86
Quercetin	C _15_H _10_O _7_	301.0354	Negative	0.1–10	y=0.0509x+0.00467	0.01/0.03	0.9978	96.41
Salicylic acid	C _7_H _6_O _3_	137.0244	Negative	0.3–10	y=0.0361x+0.00245	0.01/0.03	0.9982	92.88
Naringenin	C _15_H _12_O _5_	271.0612	Negative	0.1–10	y=0.0281x+0.00182	0.01/0.03	0.9995	86.65
Luteolin	C _15_H _10_O _6_	285.0405	Negative	0.1–10	y=0.117x+0.00848	0.01/0.03	0.9981	96.98
Apigenin	C _15_H _10_O _5_	269.0456	Negative	0.3–10	y=0.104x+0.0199	0.01/0.03	0.9998	81.55
Hispidulin	C _16_H _12_O _6_	301.0707	Positive	0.05–10	y=0.02614x+0.0003114	0.01/0.03	0.9993	98.36
Chrysin	C _15_H _10_O _4_	253.0506	Negative	0.05–7	y=0.0964x-0.0002622	0.01/0.03	0.999	87.92
Acacetin	C _16_H _12_O _5_	283.0612	Negative	0.05–7	y=0.046x+0.0001875	0.01/0.03	0.9995	87.52
Pyrogallol	C _6_H _6_O _3_	125.0244	Negative	0.5–10	y=0.5283x-0.06866	0.35/1.17	0.9954	98.38
Dihydrocaffeic acid	C _18_H _23_NO _7_	366.1547	Negative	0.5–10	y=0.06102x-0.00989	0.14/0.46	0.999	100.77
Chrysoeriol	C _16_H _12_O _6_	299.0561	Negative	0.5–10	y=0.1023x-0.002224	0.15/0.5	0.9974	96.42
Sclareol	C _20_H _36_O _2_	273.2575	Positive	0.5–10	y=0.3233x+0.0004172	0.12/0.39	0.9984	100.59

a Dihydrocapsaicin used as an internal standard,

b LOD: limit of detection; LOQ: limit of quantification.

Figure S1 The cell viability of CH: hexane extract of cone, CE: ethanol extract of cone, CD: dichloromethane extract of cone, CA: acetone extract of cone, CEW: ethanol-water (1:1) extract of cone, CEO: essential oil of cone, RH: hexane extract of resin, RE: ethanol extract of resin, REO: essential oil of resin samples and doxorubicin in HepG2 cell line.

Figure S2 The cell viability of CH: hexane extract of cone, CE: ethanol extract of cone, CD: dichloromethane extract of cone, CA: acetone extract of cone, CEW: ethanol-water (1:1) extract of cone, CEO: essential oil of cone, RH: hexane extract of resin, RE: ethanol extract of resin, REO: essential oil of resin samples and doxorubicin in PANC-1 cell line.

Figure S3 The cell viability of CH: hexane extract of cone, CE: ethanol extract of cone, CD: dichloromethane extract of cone, CA: acetone extract of cone, CEW: ethanol-water (1:1) extract of cone, CEO: essential oil of cone, RH: hexane extract of resin, RE: ethanol extract of resin, REO: essential oil of resin samples and doxorubicin in PC-3 cell line.

Figure S4 The cell viability of CH: hexane extract of cone, CE: ethanol extract of cone, CD: dichloromethane extract of cone, CA: acetone extract of cone, CEW: ethanol-water (1:1) extract of cone, CEO: essential oil of cone, RH: hexane extract of resin, RE: ethanol extract of resin, REO: essential oil of resin samples and doxorubicin in A549 cell line.

Figure S5 The cell viability of CH: hexane extract of cone, CE: ethanol extract of cone, CD: dichloromethane extract of cone, CA: acetone extract of cone, CEW: ethanol-water (1:1) extract of cone, CEO: essential oil of cone, RH: hexane extract of resin, RE: ethanol extract of resin, REO: essential oil of resin samples and doxorubicin in CCD-34Lu cell line.

Figure S6 The cell viability of CH: hexane extract of cone, CE: ethanol extract of cone, CD: dichloromethane extract of cone, CA: acetone extract of cone, CEW: ethanol-water (1:1) extract of cone, CEO: essential oil of cone, RH: hexane extract of resin, RE: ethanol extract of resin, REO: essential oil of resin samples and doxorubicin in MDA-MB 231 cell line.

Figure S7 The IC_50_ results with R^2^ values of CH: hexane extract of cone, CE: ethanol extract of cone, CD: dichloromethane extract of cone, CA: acetone extract of cone, CEW: ethanol-water (1:1) extract of cone, CEO: essential oil of cone, RH: hexane extract of resin, RE: ethanol extract of resin, REO: essential oil of resin samples and doxorubicin in MDA-MB 231 cell line.

Figure S8 The IC_50_ results with R^2^ values of CH: hexane extract of cone, CE: ethanol extract of cone, CD: dichloromethane extract of cone, CA: acetone extract of cone, CEW: ethanol-water (1:1) extract of cone, CEO: essential oil of cone, RH: hexane extract of resin, RE: ethanol extract of resin, REO: essential oil of resin samples and doxorubicin in HepG2 cell line.

Figure S9 The IC_50_ results with R^2^ values of CH: hexane extract of cone, CE: ethanol extract of cone, CD: dichloromethane extract of cone, CA: acetone extract of cone, CEW: ethanol-water (1:1) extract of cone, CEO: essential oil of cone, RH: hexane extract of resin, RE: ethanol extract of resin, REO: essential oil of resin samples and doxorubicin in PANC-1 cell line.

Figure S10 The IC_50_ results with R^2^ values of CH: hexane extract of cone, CE: ethanol extract of cone, CD: dichloromethane extract of cone, CA: acetone extract of cone, CEW: ethanol-water (1:1) extract of cone, CEO: essential oil of cone, RH: hexane extract of resin, RE: ethanol extract of resin, REO: essential oil of resin samples and doxorubicin in PC-3 cell line.

Figure S11 The IC_50_ results with R^2^ values of CH: hexane extract of cone, CE: ethanol extract of cone, CD: dichloromethane extract of cone, CA: acetone extract of cone, CEW: ethanol-water (1:1) extract of cone, CEO: essential oil of cone, RH: hexane extract of resin, RE: ethanol extract of resin, REO: essential oil of resin samples and doxorubicin in A549 cell line.

Figure S12 The IC_50_ results with R^2^ values of CH: hexane extract of cone, CE: ethanol extract of cone, CD: dichloromethane extract of cone, CA: acetone extract of cone, CEW: ethanol-water (1:1) extract of cone, CEO: essential oil of cone, RH: hexane extract of resin, RE: ethanol extract of resin, REO: essential oil of resin samples and doxorubicin in CCD-34Lu cell line.

Figure S13 Embryos removed from treated SPF-ECE’s
**a)** Positive control (only virus), **b)** Negative control (untreated ECE), c**)** Enfluvir (5 μg/g), **d)** Enfluvir (10 μg/g), **e)** CEO (0.5% v/v), **f)** CEO (5% v/v), **g)** CH (5 μg/g), **h)** CH (10 μg/g), **i)** CD (5 μg/g), **j)** CD (10 μg/g), **k)** CA (5 μg/g), **l)** CA (10 μg/g), **m)** CE (5 μg/g), **n)** CE (10 μg/g), **o)** CEW (5 μg/g), **p)** CEW (10 μg/g), **r)** RE (5 μg/g), **s)** RE (10 μg/g), **t)** RH (5 μg/g), **u)** RH (10 μg/g), **v)** REO (5 μg/g), **y)** REO (10 μg/g).

## Figures and Tables

**Figure:**
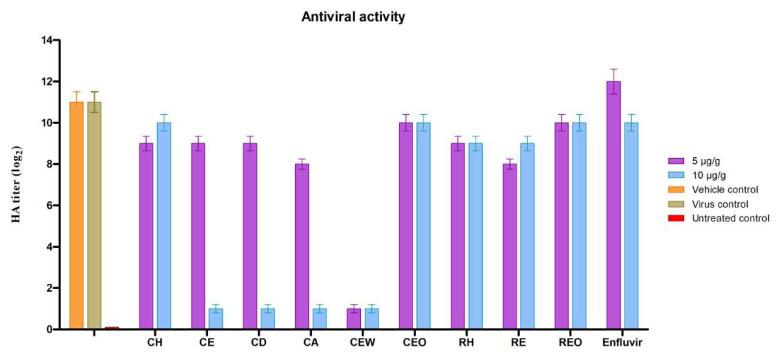
HA titers of SPF-ECE embryos after incubation with CH: hexane extract of cone, CE: ethanol extract of cone, CD: dichloromethane extract of cone, CA: acetone extract of cone, CEW: ethanol-water (1:1) extract of cone, CEO: essential oil of cone, RH: hexane extract of resin, RE: ethanol extract of resin, REO: essential oil of resin samples, enfluvir and control groups on IBV after 48 h of incubation.

**Table 1:** The essential oil composition (%) of resin from *A. nordmanniana* subsp. *bornmulleriana* and cone from *Abies nordmanniana* subsp. *equi-trojani*.

RRI^a^	Compounds	Cone^b^	Resin^b^	Identification methods^c^
1023	*α*-pinene	28.79	-	GC-FID, GC-MS
1190	*β*-pinene	3.55	-	GC-FID, GC-MS
1197	Limonene	33.50	-	GC-FID, GC-MS
1557	1-octanol	1.16	-	GC-FID, GC-MS
1647	*trans*-pinocarveol	2.86	2.24	GC-FID, GC-MS
1713	*cis*-verbenol	3.17	-	GC-FID, GC-MS
1692	*α*-terpineol	1.96	7.72	GC-FID, GC-MS
1699	Verbenone	3.27	18.16	GC-FID, GC-MS
1728	Carvone	3.03	-	GC-FID, GC-MS
1783	Myrtenol	1.49	6.88	GC-FID, GC-MS
1829	*trans* (+)-carveol	3.15	8.11	GC-FID, GC-MS
1844	*p*-cymen-8-ol	1.31	7.04	GC-FID, GC-MS
1859	*cis-*carveol	1.74	-	GC-FID, GC-MS
1967	Caryophyllene oxide	1.19	5.90	GC-FID, GC-MS
2265	Limonene glycol	5.17	-	GC-FID, GC-MS
2312	*trans*-sobreol	2.98	2.17	GC-FID, GC-MS
1960	2,5-diethylfuran	-	10.79	GC-FID, GC-MS
2185	*Z*-3-pinen-2-ol	-	1.90	GC-FID, GC-MS
2118	*p*-mentha-1,5-dien-8-ol	-	5.7	GC-FID, GC-MS
2190	*p*-mentha-1,7-dien-8-ol	-	4.05	GC-FID, GC-MS
2250	(E,E)-7,11,15, trimethyl-3-methylene-hexadeca-1,6,10,14-tetraene	-	9.71	GC-FID, GC-MS
2298	Nerolidol	-	3.39	GC-FID, GC-MS
2294	Spiro[alpha,alpha-dimethylcyclohexane-1-methanol-1,2-oxirane] isomer	-	2.59	GC-FID, GC-MS
2386	15-crown-5	-	1.54	GC-FID, GC-MS
2410	21-crown-7	-	2.01	GC-FID, GC-MS
	Undefined	1.67	0.1	GC-FID, GC-MS

a Relative retention indices were calculated against *n*-alkanes (Supelco 49452-U) on the HP-Innovax (19091N-116) capillary column.

b % values were calculated from the FID data.

c GC and GC-MS identifications based on the basis of computer matching of the mass spectra of the peaks with the Nist-Wiley and Arge-Far essential oil libraries.

**Table 2:** Fatty acid composition (%^a^) of hexane extracts of resin from *A. nordmanniana* subsp. *bornmulleriana* and cone from *Abies nordmanniana* subsp. *equi-trojani*.

Fatty acids^b^	Resin	Cone
16:0	20.81	20.83
18:0	14.24	12.49
18:1 n9c	41.00	55.74
18:2 n6c	15.66	10.94
22:0	8.28	-

a Percentage of total fatty acids.

b Carbons and double bond numbers of fatty acids.

**Table 3:** IC_50_ values for cytotoxic activities of *Abies nordmanniana* subsp. on different cell lines in μg/mL.

	CCD-34Lu	MDA-MB-231	PANC-1	PC-3	HepG2	A549
**CH**	>50	>50	48.6 ± 5.28	>50	36.67±3.68	>50
**CE**	>50	>50	>50	>50	>50	>50
**CD**	>50	>50	>50	>50	29.08 ± 5.61	>50
**CA**	>50	>50	>50	>50	43.95 ± 0.3	>50
**CEW**	>50	>50	>50	>50	32.53 ± 2.25	>50
**CEO**	>50	>50	>50	>50	>50	>50
**RH**	>50	>50	>50	>50	17.725± 2.09	>50
**RE**	>50	>50	>50	>50	>50	>50
**REO**	>50	>50	>50	>50	>50	>50
**Doxorubicin**	8.012 ± 0.01	15.22 ± 0.02	5.207 ± 0.01	4.23 ± 0.02	36.85 ± 0.02	1.21 ± 0.01

* CH: hexane extract of cone, CE: ethanol extract of cone, CD: dichloromethane extract of cone, CA: acetone extract of cone, CEW: ethanol-water (1:1) extract of cone, CEO: Essential oils of cone, RH: hexane extract of resin, RE: ethanol extract of resin, REO: essential oils of resin.

**Table 4:** Phenolic and flavonoid contents of (mg/kg extract) resin ethanol extract, cone ethanol, ethanol-water (1:1), dichloromethane, and acetone extracts of *Abies nordmanniana* subsp.

	RE	CEW	CE	CD	CA*	U % **
Ascorbic acid	71.46	2508.50	36.27	18.98	56.54	3.94
Fumaric acid	57050.00	<LOD	<LOD	<LOD	<LOD	2.88
(−)-Epicatechin gallate	<LOD	3.33	<LOD	<LOD	<LOD	3.05
Verbascoside	3.22	<LOD	<LOD	6.60	1.13	2.93
Chicoric acid	<LOD	10.83	7.18	<LOD	11.86	2.28
Caffeic acid	40.12	8.00	10.14	38.10	19.48	3.74
(+)-*trans* taxifolin	<LOD	5.56	4.11	0.15	6.07	3.35
Vanilic acid	<LOD	1691.89	1152.93	<LOD	1554.355	3.49
Luteolin 7-glucoside	7.88	<LOD	<LOD	9.19	<LOD	4.14
Rutin	<LOD	47.33	1.53	1.20	1.69	3.07
Rosmarinic acid	158.69	32.78	24.05	130.13	35.44	3.77
Hyperoside	<LOD	263.44	51.78	<LOD	44.12	3.46
Dihydrokaempferol	<LOD	21.72	19.73	1.54	24.28	2.86
Apigenin 7-glucoside	1.61	5.00	0.49	1.84	0.92	3.59
Quercitrin	<LOD	283.28	59.73	<LOD	49.76	3.78
Myricetin	<LOD	18.22	4.33	<LOD	6.35	4.18
Quercetin	1.25	63.44	51.45	0.30	65.51	2.95
Salicylic acid	282.45	259.89	270.68	103.91	352.66	1.89
Naringenin	3.04	37.44	37.92	9.79	50.96	4.20
Apigenin	5.55	2.61	1.75	0.41	2.12	2.87
Hispidulin	<LOD	1.44	1.75	<LOD	1.69	3.41

* RE: Ethanol extract of resin (*A. nordmanniana* subsp. *bornmulleriana*), CEW: Ethanol-water (1:1) extract of cone, CE: Ethanol extract of cone, CD: Dichloromethane extract of cone, CA: Acetone extract of cone (*A. nordmanniana* subsp. *equi-trojani*),

** U%: The percent relative uncertainties of the reported compounds.

**Table 5:** The virucidal effects of *Abies nordmanniana* subsp. extracts on IBV after 48 h of incubation.

Sample*	Concentration μg/g	Egg mortality	Mortality (%)	HA titer (mean)	HA titer (log_2_) (mean)
**Positive control (only virus)**		1/4	25	2048	11
**Negative control (untreated ECE)**		0/4	0	0	0
**Vehicle control (5% DMSO)**		0/4	0	2048	11
**CH**	5 μg/g	1/4	25	512	9
	10 μg/g	0/4	0	1024	10
**CE**	5 μg/g	1/4	25	512	9
	10 μg/g	1/4	0	2	1
**CD**	5 μg/g	0/4	0	512	9
	10 μg/g	0/4	0	2	1
**CA**	5 μg/g	0/4	0	256	8
	10 μg/g	1/4	25	2	1
**CEW**	5 μg/g	0/4	0	2	1
	10 μg/g	3/4	75	2	1
**CEO**	0.5% v/v	0/4	0	1024	10
	5% v/v	1/4	25	1024	10
**RH**	5 μg/g	1/4	25	512	9
	10 μg/g	0/4	0	512	9
**RE**	5 μg/g	2/4	50	256	8
	10 μg/g	1/4	25	512	9
**REO**	5 μg/g	1/4	25	1024	10
	10 μg/g	1/4	25	1024	10
**Enfluvir**	5 μg/g	1/4	25	4096	12
	10 μg/g	0/4	0	1024	10

* CH: hexane extract of cone, CE: ethanol extract of cone, CD: dichloromethane extract of cone, CA: acetone extract of cone, CEW: ethanol-water (1:1) extract of cone (*A. nordmanniana* subsp. *equi-trojani*), CEO: Essential oils of cone, RH: hexane extract of resin, RE: ethanol extract of resin, REO: essential oils of resin (*A. nordmanniana* subsp. *bornmulleriana*).
