# Supplementary figures and images for: Antiviral and cytotoxic activities and chemical profiles of two species of Abies nordmanniana from Türkiye
Source: Turk J Chem. 2024 Mar 11;48(3):436–47. doi: 10.55730/1300-0527.3670 (PMC11265925; doi:10.55730/1300-0527.3670)

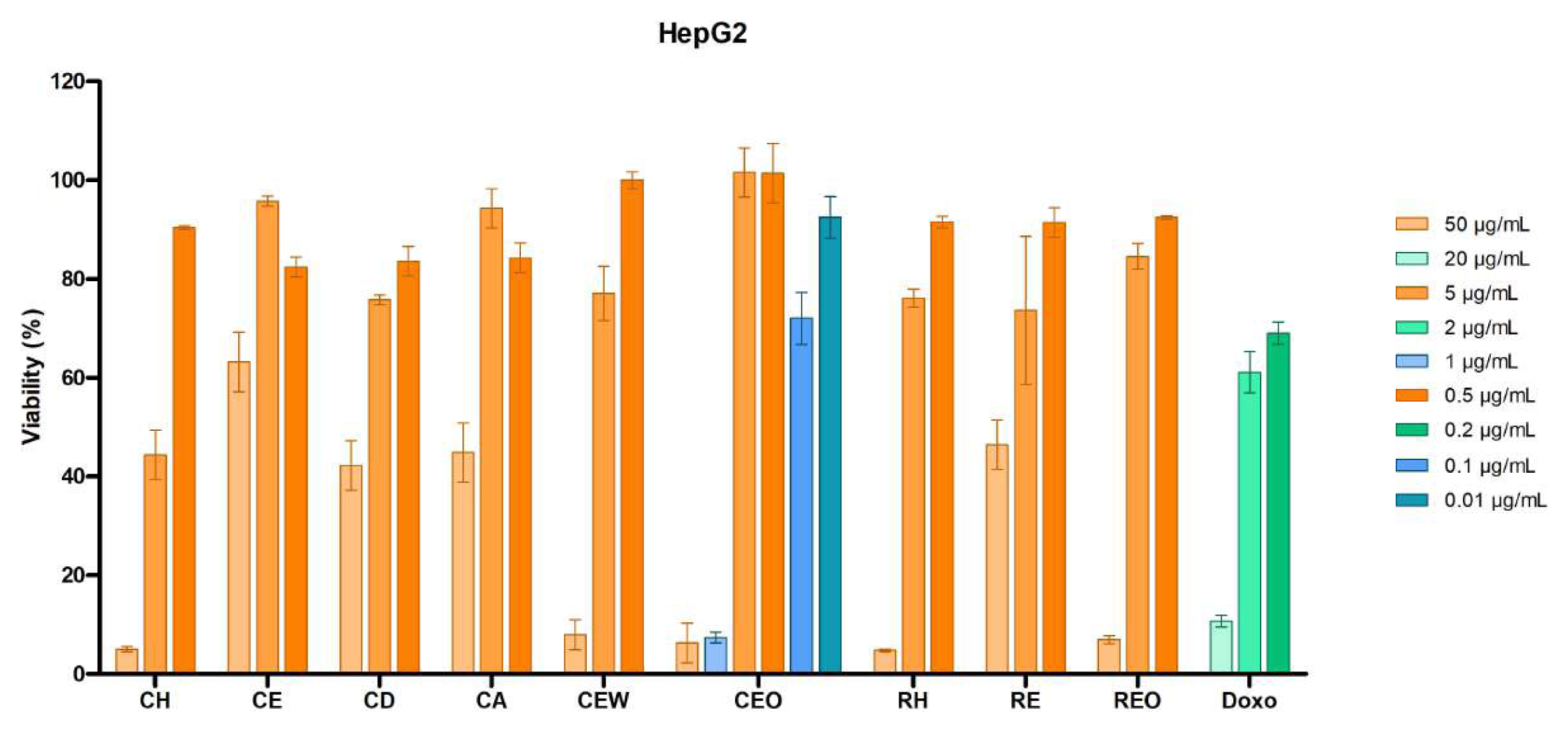

Supplement: Figure S1 — The cell viability of CH: hexane extract of cone, CE: ethanol extract of cone, CD: dichloromethane extract of cone, CA: acetone extract of cone, CEW: ethanol-water (1:1) extract of cone, CEO: essential oil of cone, RH: hexane extract of resin, RE: ethanol extract of resin, REO: essential oil of resin samples and doxorubicin in HepG2 cell line. [file tjc-48-03-436s1.tif]

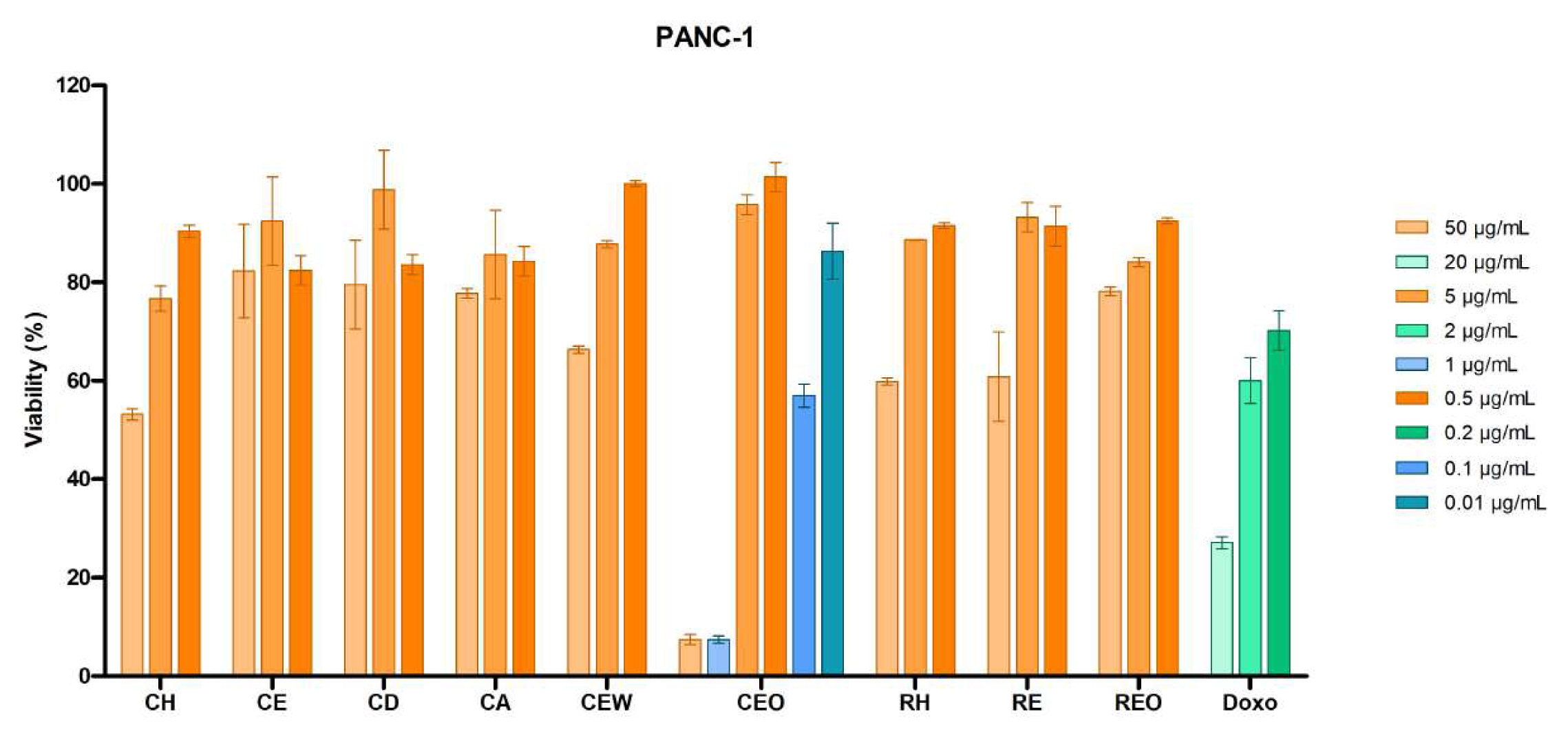

Supplement: Figure S2 — The cell viability of CH: hexane extract of cone, CE: ethanol extract of cone, CD: dichloromethane extract of cone, CA: acetone extract of cone, CEW: ethanol-water (1:1) extract of cone, CEO: essential oil of cone, RH: hexane extract of resin, RE: ethanol extract of resin, REO: essential oil of resin samples and doxorubicin in PANC-1 cell line. [file tjc-48-03-436s2.tif]

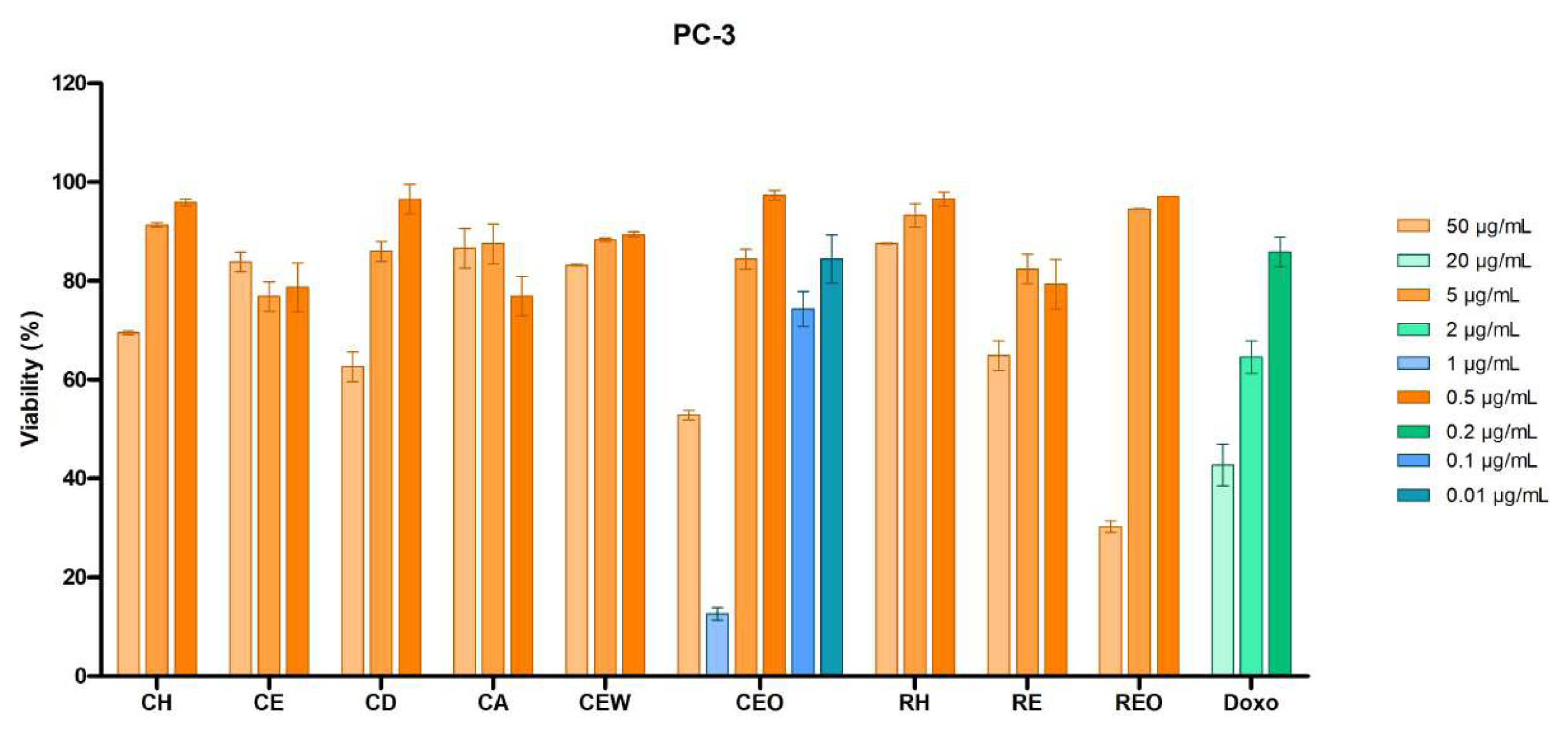

Supplement: Figure S3 — The cell viability of CH: hexane extract of cone, CE: ethanol extract of cone, CD: dichloromethane extract of cone, CA: acetone extract of cone, CEW: ethanol-water (1:1) extract of cone, CEO: essential oil of cone, RH: hexane extract of resin, RE: ethanol extract of resin, REO: essential oil of resin samples and doxorubicin in PC-3 cell line. [file tjc-48-03-436s3.tif]

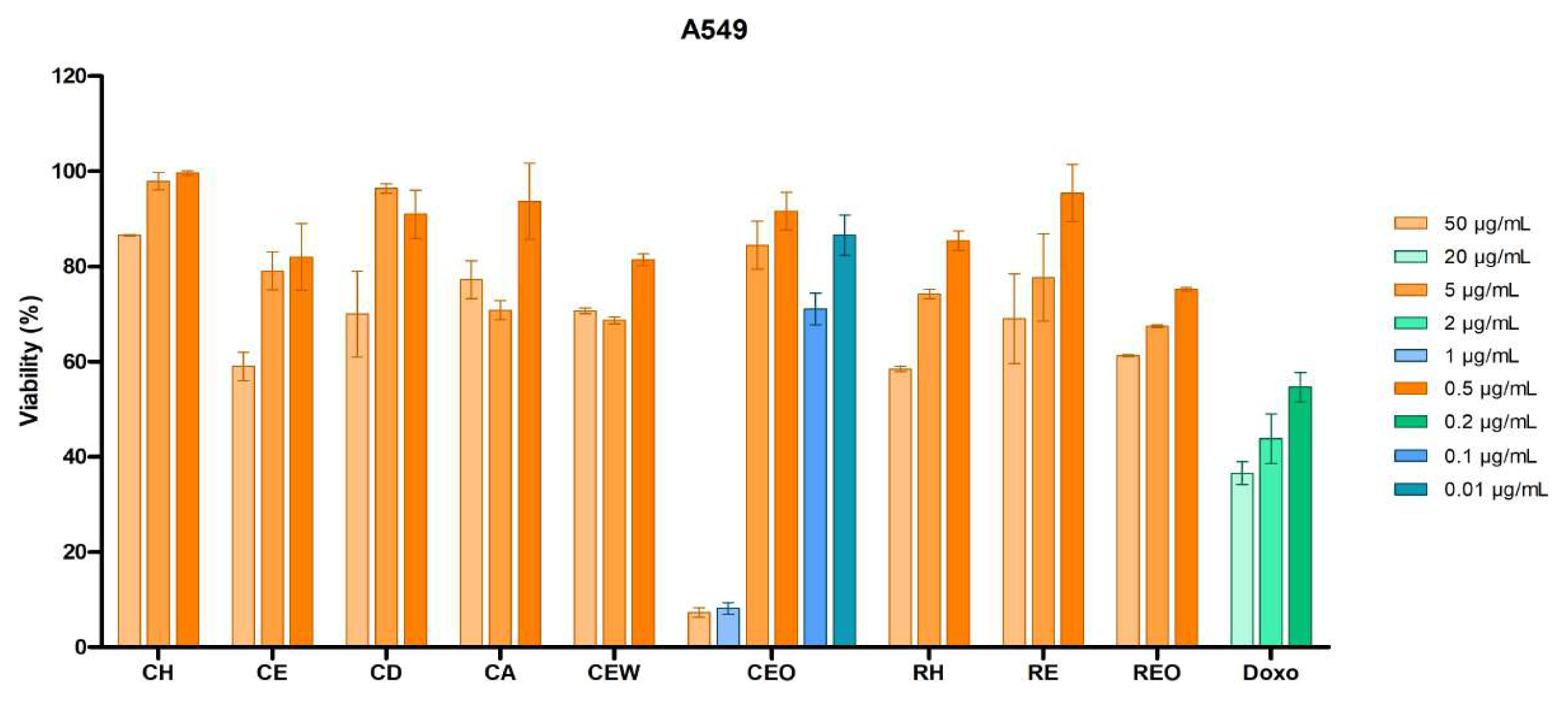

Supplement: Figure S4 — The cell viability of CH: hexane extract of cone, CE: ethanol extract of cone, CD: dichloromethane extract of cone, CA: acetone extract of cone, CEW: ethanol-water (1:1) extract of cone, CEO: essential oil of cone, RH: hexane extract of resin, RE: ethanol extract of resin, REO: essential oil of resin samples and doxorubicin in A549 cell line. [file tjc-48-03-436s4.tif]

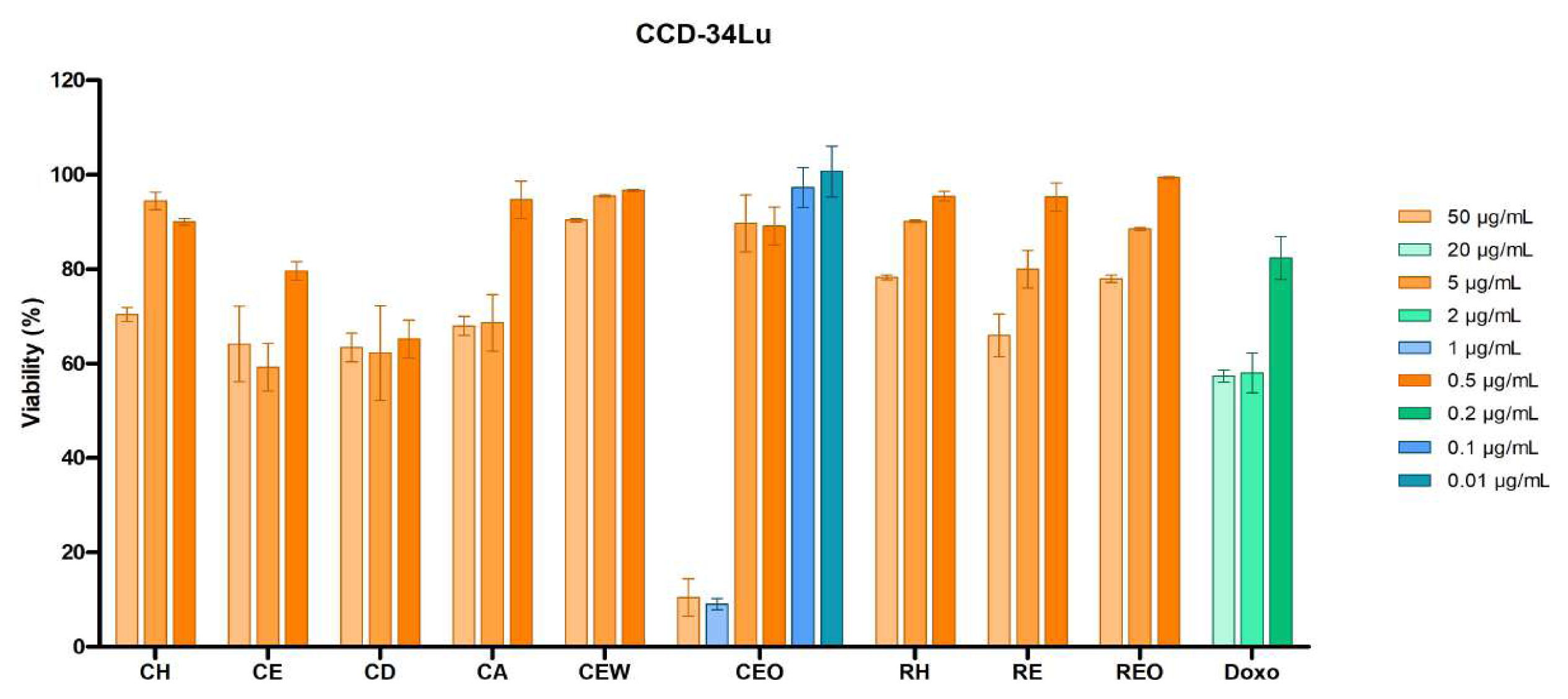

Supplement: Figure S5 — The cell viability of CH: hexane extract of cone, CE: ethanol extract of cone, CD: dichloromethane extract of cone, CA: acetone extract of cone, CEW: ethanol-water (1:1) extract of cone, CEO: essential oil of cone, RH: hexane extract of resin, RE: ethanol extract of resin, REO: essential oil of resin samples and doxorubicin in CCD-34Lu cell line. [file tjc-48-03-436s5.tif]

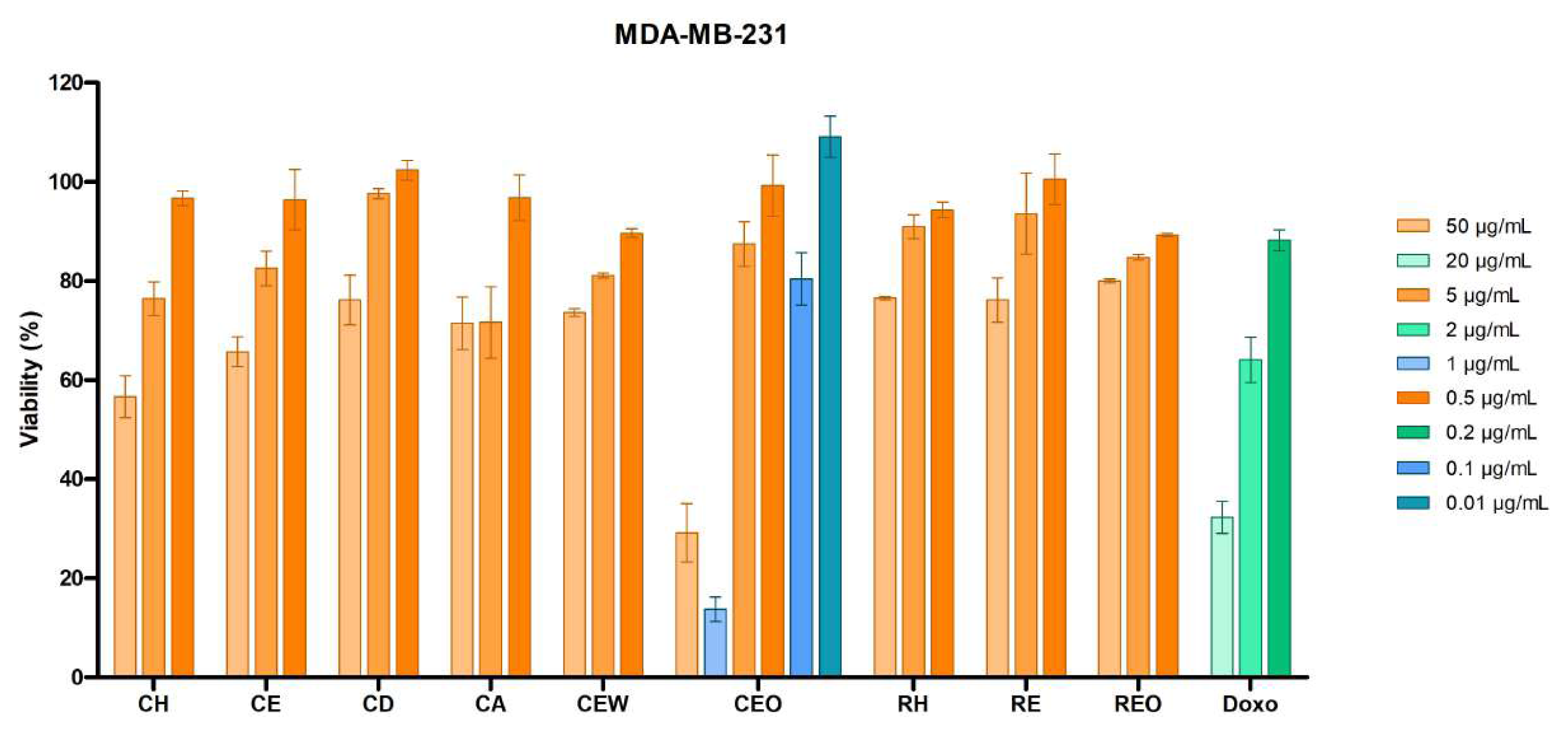

Supplement: Figure S6 — The cell viability of CH: hexane extract of cone, CE: ethanol extract of cone, CD: dichloromethane extract of cone, CA: acetone extract of cone, CEW: ethanol-water (1:1) extract of cone, CEO: essential oil of cone, RH: hexane extract of resin, RE: ethanol extract of resin, REO: essential oil of resin samples and doxorubicin in MDA-MB 231 cell line. [file tjc-48-03-436s6.tif]

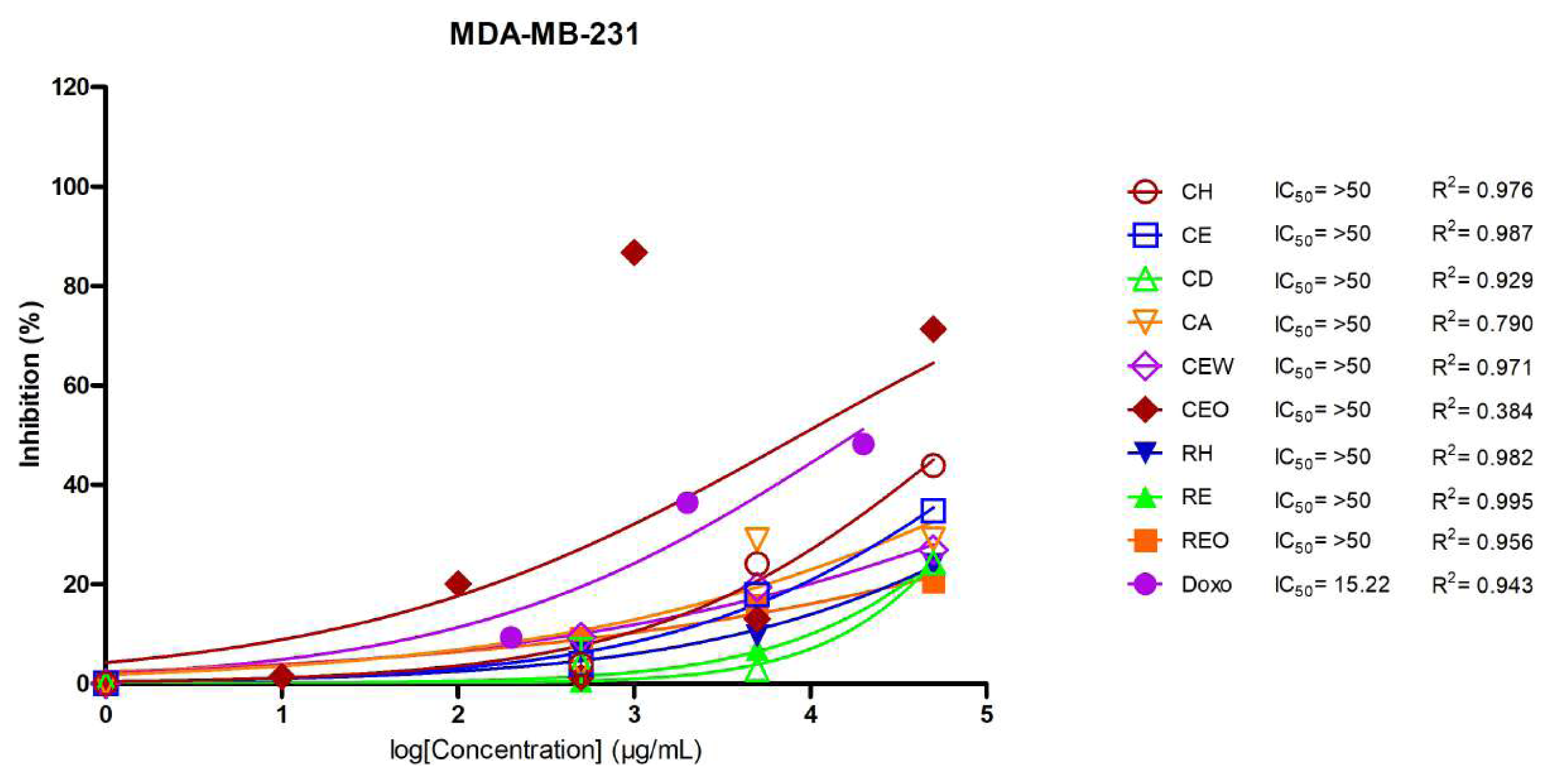

Supplement: Figure S7 — The IC50 results with R2 values of CH: hexane extract of cone, CE: ethanol extract of cone, CD: dichloromethane extract of cone, CA: acetone extract of cone, CEW: ethanol-water (1:1) extract of cone, CEO: essential oil of cone, RH: hexane extract of resin, RE: ethanol extract of resin, REO: essential oil of resin samples and doxorubicin in MDA-MB 231 cell line. [file tjc-48-03-436s7.tif]

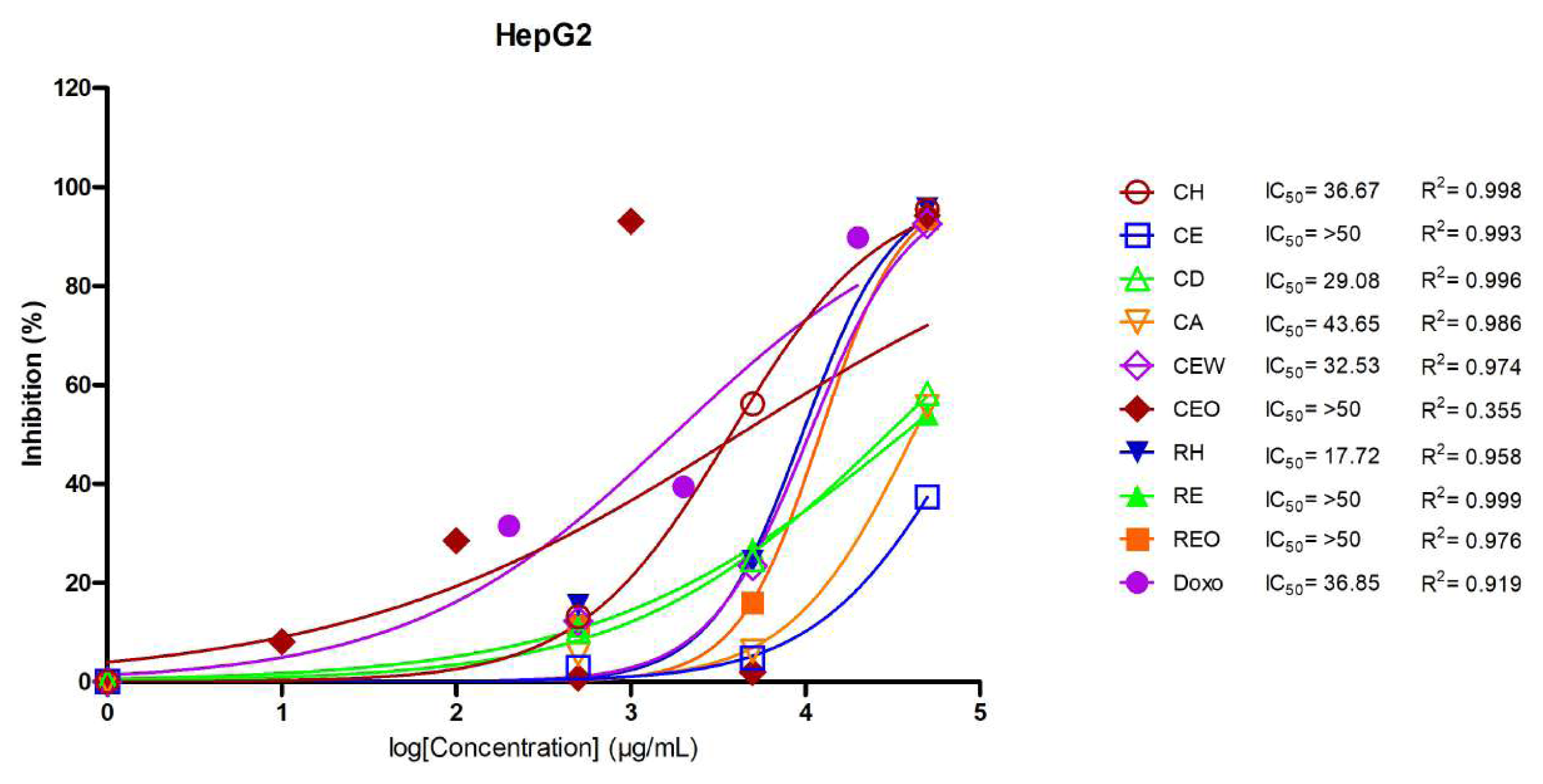

Supplement: Figure S8 — The IC50 results with R2 values of CH: hexane extract of cone, CE: ethanol extract of cone, CD: dichloromethane extract of cone, CA: acetone extract of cone, CEW: ethanol-water (1:1) extract of cone, CEO: essential oil of cone, RH: hexane extract of resin, RE: ethanol extract of resin, REO: essential oil of resin samples and doxorubicin in HepG2 cell line. [file tjc-48-03-436s8.tif]

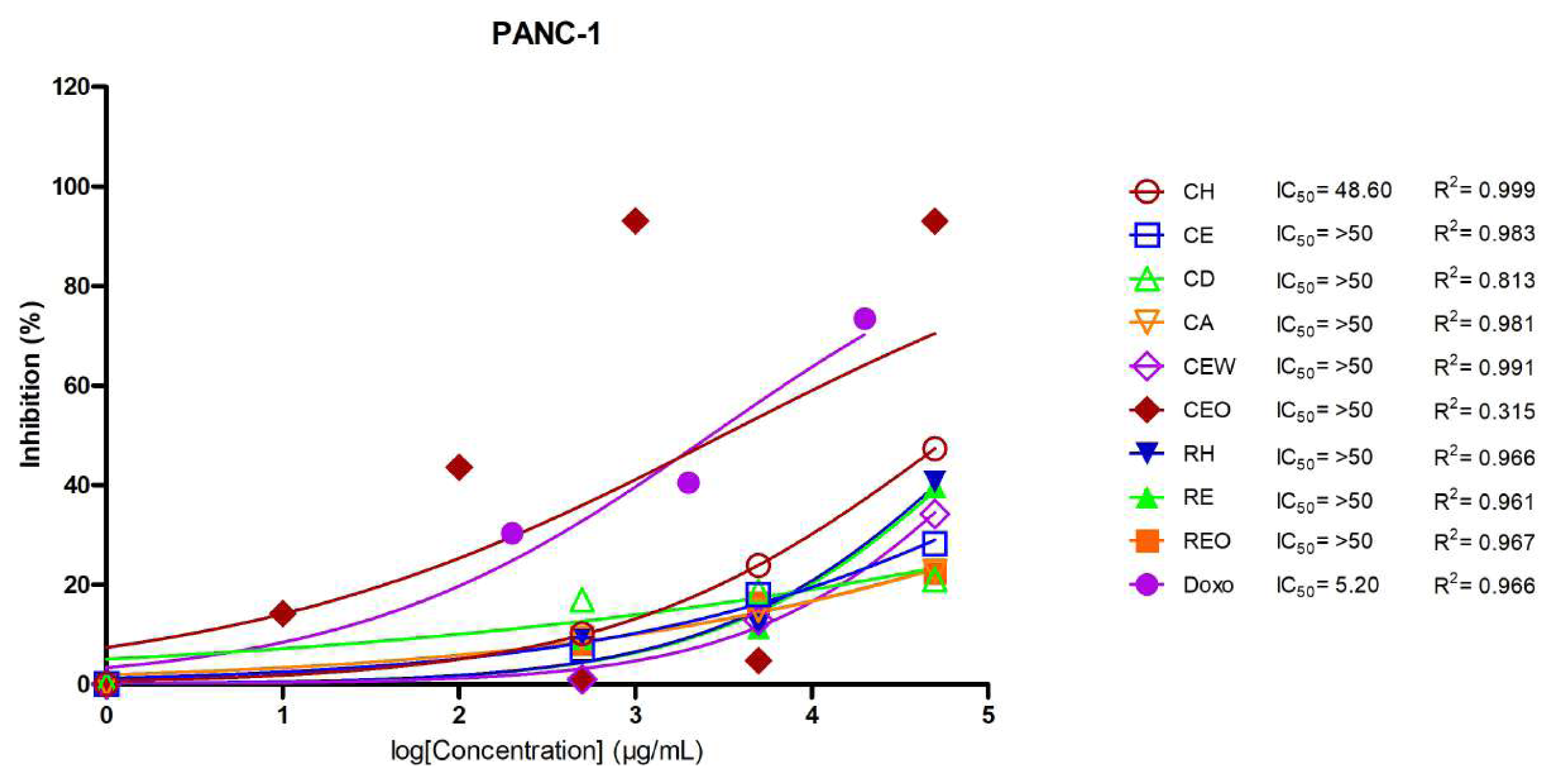

Supplement: Figure S9 — The IC50 results with R2 values of CH: hexane extract of cone, CE: ethanol extract of cone, CD: dichloromethane extract of cone, CA: acetone extract of cone, CEW: ethanol-water (1:1) extract of cone, CEO: essential oil of cone, RH: hexane extract of resin, RE: ethanol extract of resin, REO: essential oil of resin samples and doxorubicin in PANC-1 cell line. [file tjc-48-03-436s9.tif]

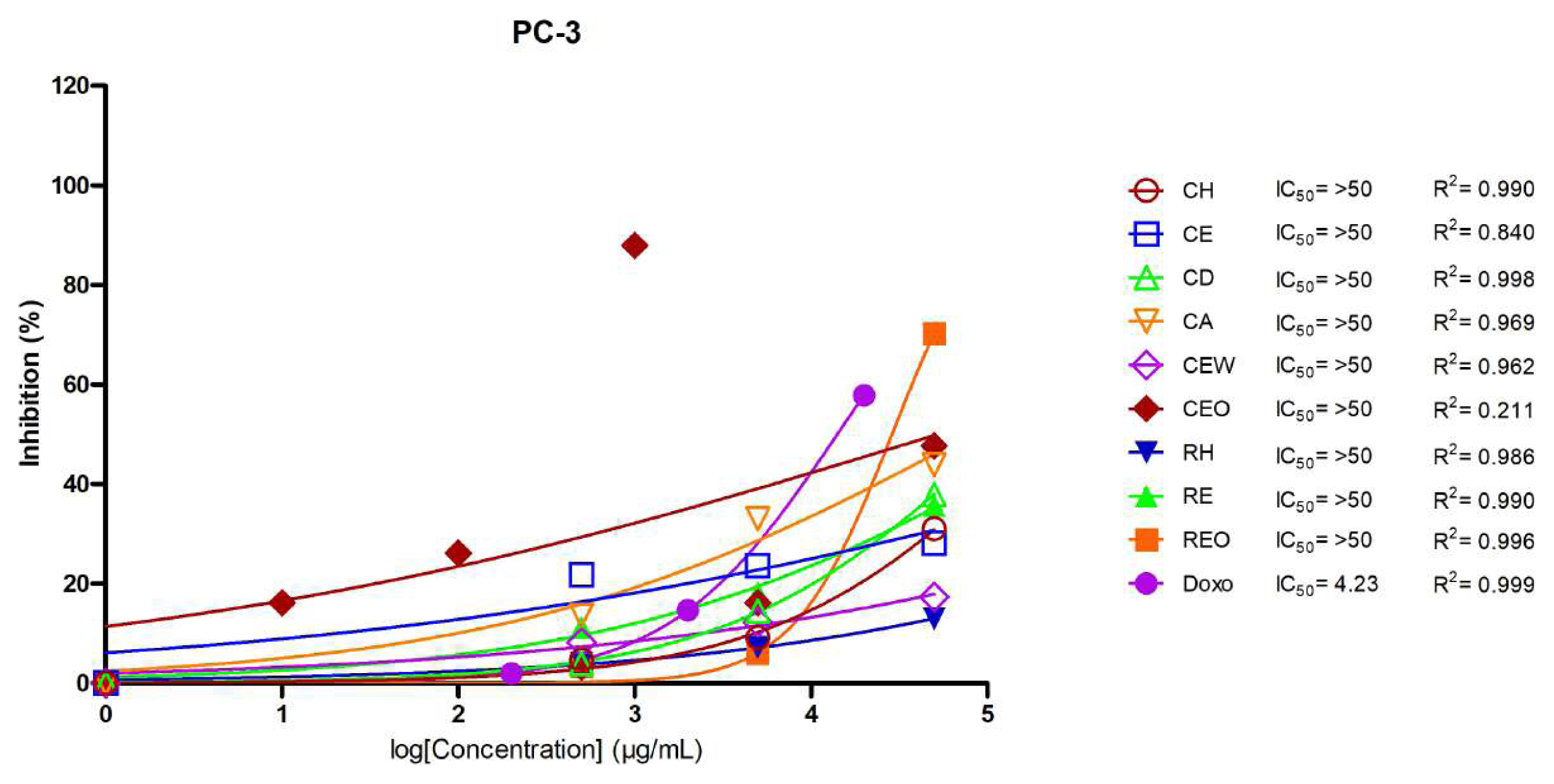

Supplement: Figure S10 — The IC50 results with R2 values of CH: hexane extract of cone, CE: ethanol extract of cone, CD: dichloromethane extract of cone, CA: acetone extract of cone, CEW: ethanol-water (1:1) extract of cone, CEO: essential oil of cone, RH: hexane extract of resin, RE: ethanol extract of resin, REO: essential oil of resin samples and doxorubicin in PC-3 cell line. [file tjc-48-03-436s10.tif]

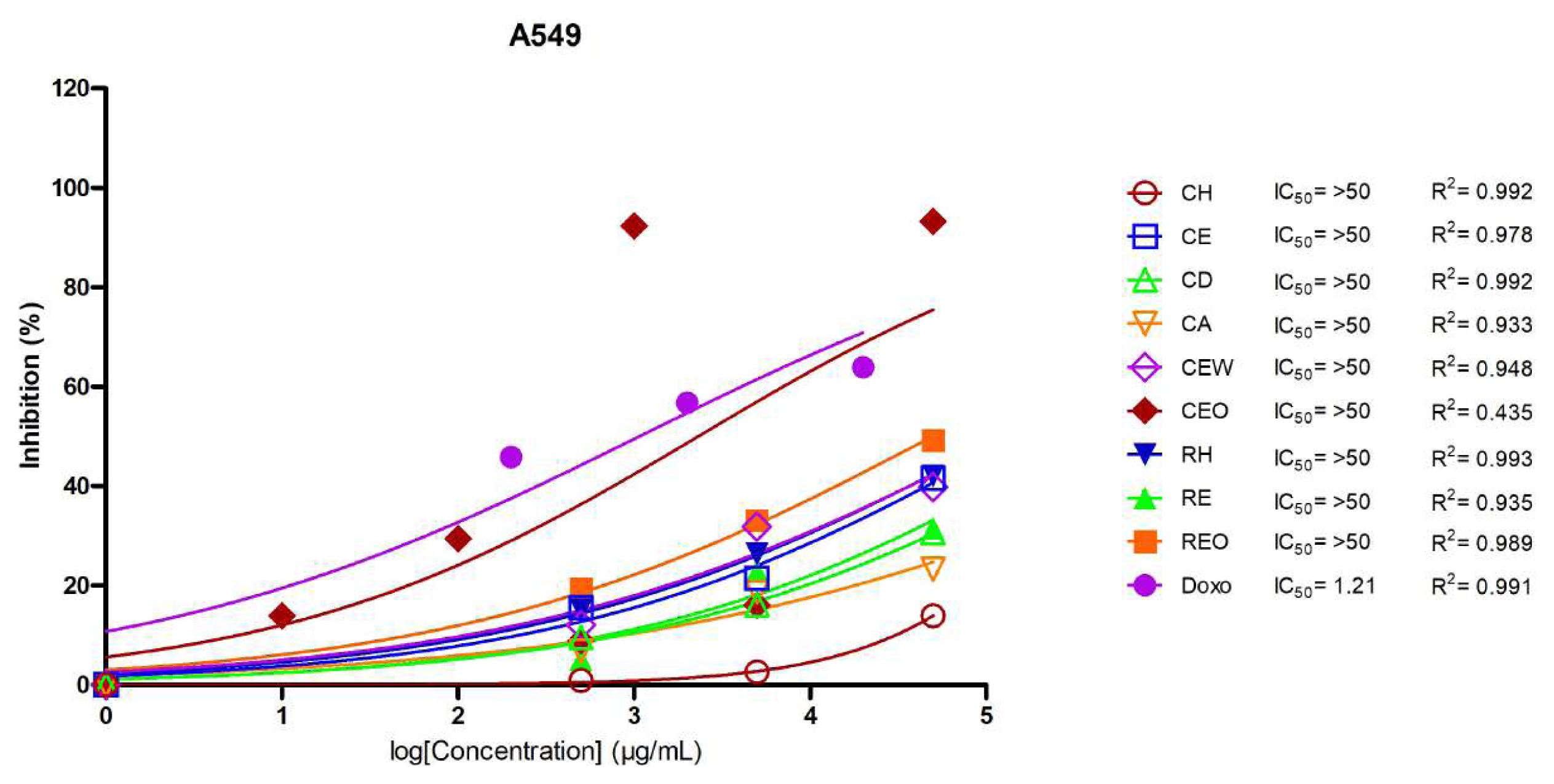

Supplement: Figure S11 — The IC50 results with R2 values of CH: hexane extract of cone, CE: ethanol extract of cone, CD: dichloromethane extract of cone, CA: acetone extract of cone, CEW: ethanol-water (1:1) extract of cone, CEO: essential oil of cone, RH: hexane extract of resin, RE: ethanol extract of resin, REO: essential oil of resin samples and doxorubicin in A549 cell line. [file tjc-48-03-436s11.tif]

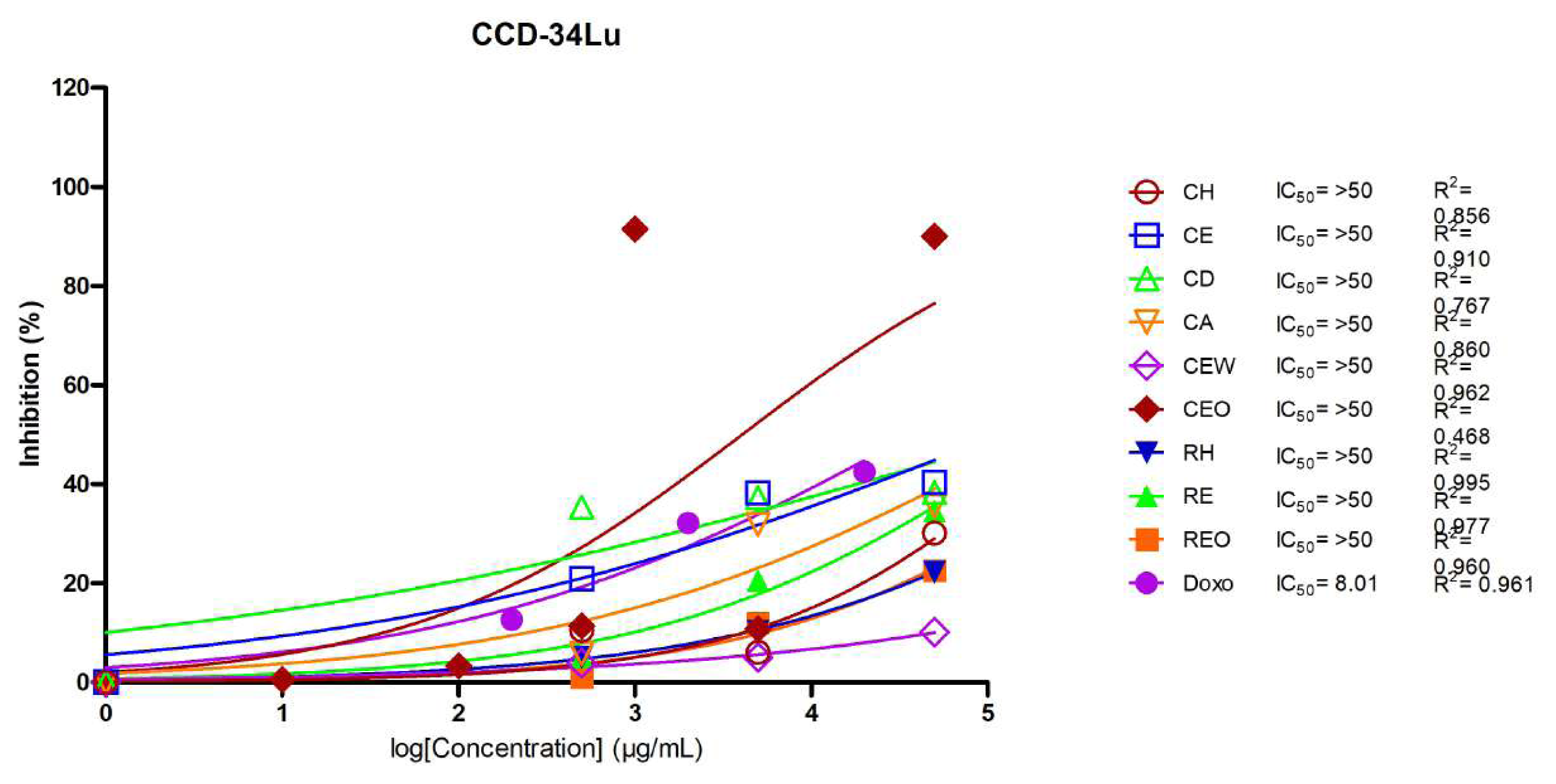

Supplement: Figure S12 — The IC50 results with R2 values of CH: hexane extract of cone, CE: ethanol extract of cone, CD: dichloromethane extract of cone, CA: acetone extract of cone, CEW: ethanol-water (1:1) extract of cone, CEO: essential oil of cone, RH: hexane extract of resin, RE: ethanol extract of resin, REO: essential oil of resin samples and doxorubicin in CCD-34Lu cell line. [file tjc-48-03-436s12.tif]

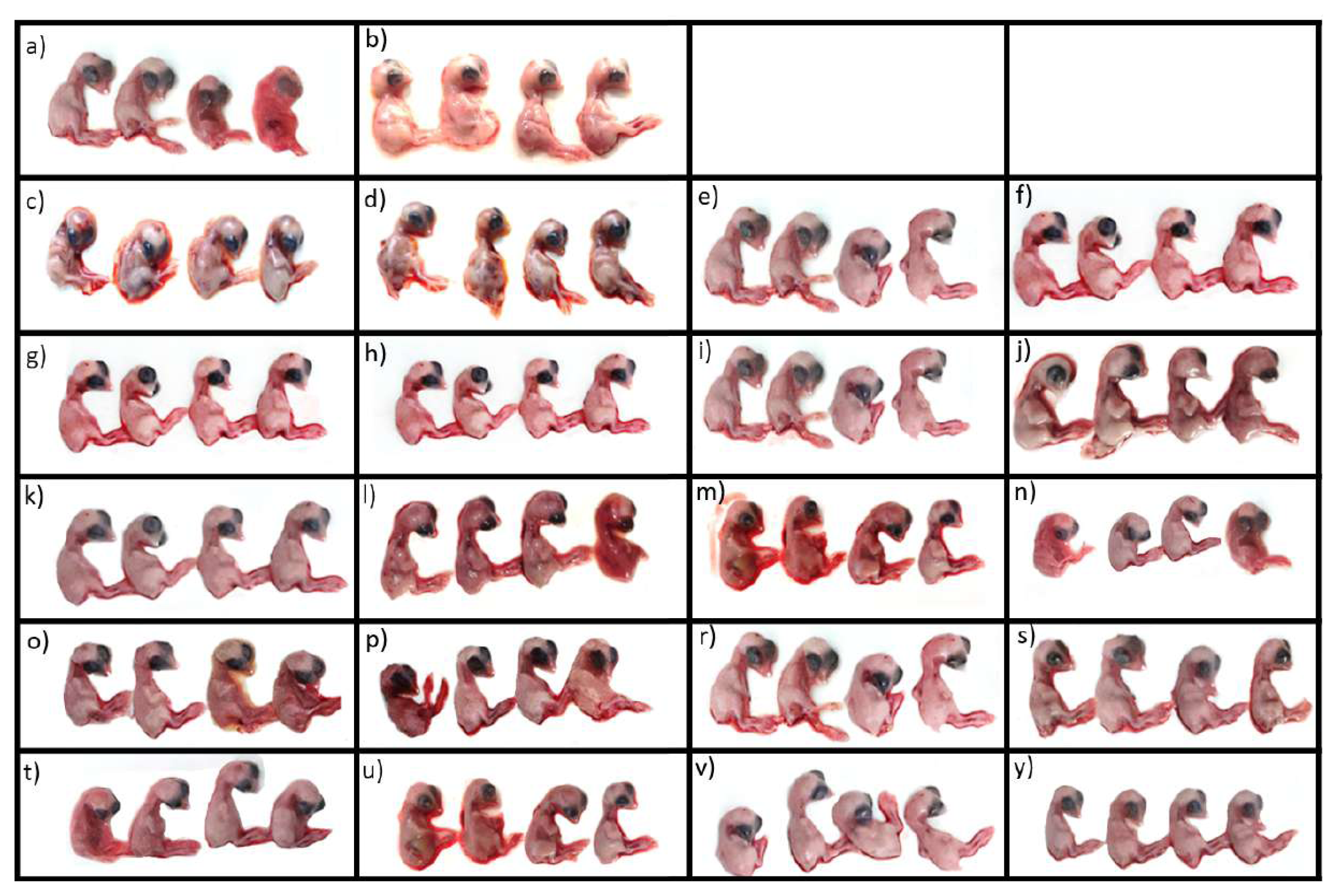

Supplement: Figure S13 — Embryos removed from treated SPF-ECE’s a) Positive control (only virus), b) Negative control (untreated ECE), c) Enfluvir (5 μg/g), d) Enfluvir (10 μg/g), e) CEO (0.5% v/v), f) CEO (5% v/v), g) CH (5 μg/g), h) CH (10 μg/g), i) CD (5 μg/g), j) CD (10 μg/g), k) CA (5 μg/g), l) CA (10 μg/g), m) CE (5 μg/g), n) CE (10 μg/g), o) CEW (5 μg/g), p) CEW (10 μg/g), r) RE (5 μg/g), s) RE (10 μg/g), t) RH (5 μg/g), u) RH (10 μg/g), v) REO (5 μg/g), y) REO (10 μg/g). [file tjc-48-03-436s13.tif]
